# Shotgun Quantitative Proteomic Analysis of Proteins Responding to Drought Stress in* Brassica rapa* L. (Inbred Line “Chiifu”)

**DOI:** 10.1155/2016/4235808

**Published:** 2016-06-21

**Authors:** Soon-Wook Kwon, Mijeong Kim, Hijin Kim, Joohyun Lee

**Affiliations:** ^1^Department of Plant Bioscience, Pusan National University, Milyang 627-706, Republic of Korea; ^2^Department of Applied Bioscience, Konkuk University, Seoul 143-701, Republic of Korea

## Abstract

Through a comparative shotgun quantitative proteomics analysis in* Brassica rapa* (inbred line Chiifu), total of 3,009 nonredundant proteins were identified with a false discovery rate of 0.01 in 3-week-old plants subjected to dehydration treatment for 0, 24, and 48 h, plants subjected to drought stress. Ribulose-bisphosphate carboxylases, chlorophyll a/b-binding protein, and light harvesting complex in photosystem II were highly abundant proteins in the leaves and accounted for 9%, 2%, and 4%, respectively, of the total identified proteins. Comparative analysis of the treatments enabled detection of 440 differentially expressed proteins during dehydration. The results of clustering analysis, gene ontology (GO) enrichment analysis, and analysis of composite expression profiles of functional categories for the differentially expressed proteins indicated that drought stress reduced the levels of proteins associated with photosynthesis and increased the levels of proteins involved in catabolic processes and stress responses. We observed enhanced expression of many proteins involved in osmotic stress responses and proteins with antioxidant activities. Based on previously reported molecular functions, we propose that the following five differentially expressed proteins could provide target genes for engineering drought resistance in plants: annexin, phospholipase D delta, sDNA-binding transcriptional regulator, auxin-responsive GH3 family protein, and TRAF-like family protein.

## 1. Introduction

Drought is a widespread environmental stress that is becoming increasingly problematic for agriculture due to the effects of climate change. Drought is caused by continuous shortages in water supply due to altered precipitation patterns in cropped areas [1, 2]. In general, drought stress causes 40% of global crop yield losses annually [3] and inhibits plant growth and development [4] by reducing root expansion, root development, leaf size, and seed development [5, 6]. Water deficit directly affects photosynthesis by altering the photosynthetic systems and reducing CO_2_ availability [7]. Plant CO_2_ assimilation is reduced by stomatal closure, damaged thylakoid membranes, and disrupted activity of enzymes involved in CO_2_ fixation and adenosine triphosphate synthesis [5]. Drought stress affects ribulose-1,5-bisphosphate (RuBP) regeneration or reduces the levels of functional RuBP, which limits photosynthesis [8]. Secondary effects of drought stress include oxidative stress, which is toxic for aerobic metabolism [9].

Plants exhibit complex responses to drought stress, including changes in chloroplast metabolism and gene expression. Drought stress inhibits photosynthetic activity and causes an imbalance between light capture and light utilization [10]. Drought-mediated alterations in leaf photochemistry and photosynthetic electron transport generate potentially dangerous active oxygen species [11] and superoxide radicals [12]. Abscisic acid (ABA), a plant hormone that functions in plant growth and development, has two important roles in water stress, including regulating cellular water status to protect cell systems and inducing genes that express dehydration tolerant proteins [4]. Genetics and breeding studies suggest that the pattern of dehydration tolerance inheritance may reflect that it is conferred by quantitative trait loci (QTL). Plant stress response is a dynamic process and the drought stress response is complex in plants, thus investigation of changes in gene expression in genomic level possibly to reveal the global snapshot of the response. The development of methods to monitor gene expression at the genomic level has enabled transcriptomic and proteomic analyses of plant cell responses under stress conditions. Proteins are key components of cellular structure. Shotgun proteomics analysis can provide direct functional information by exploring broad cellular expression patterns of proteins responding to environmental or extracellular stimuli [13].

Severe drought stress can be lethal for leaf vegetables such as* B. rapa*, which is widely produced in Asia. The whole genome of Chinese cabbage (inbred line Chiifu) was sequenced [14]. This genome sequencing database provided a reference and promoted transcriptomic and proteomic studies of other genomes. Yu et al. performed tag sequencing on* B. rapa* L. ssp.* pekinensis* and identified 1,092 drought-responsive genes; 37 genes were transcription factors, 28 were involved in signal transduction, and 61 were water-sensing and osmosensing responsive genes [15]. Microarray analysis of seedlings of* B. rapa* L. ssp.* pekinensis* subjected to 48 h of drought treatment determined that 738 genes (including 56 transcription factors) were differentially expressed in response to drought [16]. The identification of abiotic stress-responsive genes using DNA and RNA analytical tools is ongoing. However, proteomic analysis of* B. rapa* subjected to abiotic stress has not been sufficiently investigated.

Gel-LC/MSMS approach is a one shotgun proteomic approach where bottom up protein identification is performed from the protein mixture [17, 18]. In Gel-LC/MSMS, proteins are first separated using 1D SDS-PAGE or IPG-IEF and then digested into peptides from the divided gel pieces. These peptides are analyzed through mass spectrometry (MSMS) combined with high-pressure liquid chromatography (HPLC) [17]. Proteins detected by MS-MS are identified by comparison with protein databases [19, 20]. Shotgun proteomic analysis is usually performed for qualitative analysis, but spectral count normalization enables relative quantitative analysis [21]. The normalization of spectral counts is a label-free approach, which utilizes the mass signal strength of the sample's proteins and the spectral counts of the protein [22].

We investigated the effects of drought on* Brassica rapa* at the protein level using shotgun proteomic analysis. Nonredundant proteins were detected in* B. rapa* seedling extracts at 0, 24, and 48 h after the start of dehydration. The relative levels and patterns of proteins were compared to provide insights into changes in proteins in response to drought and to identify candidate proteins that could confer drought resistance to other plants.

## 2. Materials and Methods

### 2.1. Plant Material and Drought Stress Treatment

*Brassica rapa* ssp. pekinensis (inbred line Chiifu) seeds were germinated in water, and then one seedling was transplanted into soil in one pot (90 mm id × 90 mm).* Brassica rapa* plants were grown for 3 weeks in a growth chamber at 25°C (16 h day/8 h night, 40–70% relative humidity) with sufficient water supply until 1 day before drought treatment. Drought treatment was performed by removing the plastic pot from the plant root mass and exposing the soil to air [16]. This drought treatment proceeded for 24 and 48 h, at which times the whole plant except root tissue was harvested. Three-week-old plants without any drought treatment served as control (or 0 h drought treatment). Triplicate biological replicates were analyzed for all the three time points (0, 24, and 48 h) for statistical analysis.

### 2.2. Protein Extraction and Trichloroacetic Acid/Acetone Precipitation

The harvested plant tissue was ground in liquid nitrogen, and proteins were extracted from the ground tissue powder by adding extraction buffer (8 M urea, 5 mM dithiothreitol (DTT), 1% lithium dodecyl sulfate (LDS), and 100 mM Tris, pH 8.5). The suspension was incubated at room temperature for 30 min with vortexing, followed by centrifugation at 14,000 ×g for 15 min. The supernatant was recovered, and extracted proteins were precipitated overnight with 20% (v/v) trichloroacetic acid (TCA), washed several times with cold acetone to remove chlorophyll, and resolubilized in 8 M urea/Tris-HCl, pH 8.5. Sample protein concentrations were determined using the 2D-Protein Quant Kit (GE Healthcare, Piscataway, NJ, USA).

### 2.3. One-Dimensional LDS-PAGE and In-Gel Trypsin Digestion

Fifty micrograms of protein samples was prepared with NuPage® LDS Sample Buffer (Invitrogen, Carlsbad, CA, USA). Proteins were separated on a 4–12% NuPAGE Novex Bis-Tris gel (Invitrogen, Carlsbad, CA, USA) and stained with Coomassie Blue G-250 (Invitrogen). After the proteins were resolved on the gel, each sample gel lane was cut out and divided into seven equal-sized pieces, and proteins were in-gel digested with trypsin using the method of Shevchenko et al. [23].

### 2.4. LC MS/MS Analysis

A nanoflow HPLC instrument (Easy nLC, Thermo Fisher Scientific, Waltham, MA, USA) was coupled online to a Q Exactive Mass Spectrometer (Thermo Fisher Scientific, San Jose, CA, USA). Analytical columns (12 cm, 75 *μ*m inner diameter) were packed in-house with Alltima C18-AQ 5 *μ*m resin. Reverse-phase chromatography was performed with a binary buffer system consisting of 0.1% formic acid (buffer A) and acetonitrile in 0.1% formic acid (buffer B). The sample was separated with a linear gradient of 3–60% buffer B at a flow rate of 250 nL/min. The total run time for the LC MS/MS was 110 min. MS data were acquired using a data-dependent top 8 method and dynamically choosing the most abundant precursor ions from the survey scan (300–2,000 Da) for higher-energy collisional dissociation (HCD) fragmentation. Dynamic exclusion duration was 60 s, and the precursor isolation window was performed with four. Survey scans were acquired at a resolution of 70,000 at* m*/*z* 200. The resolution for HCD spectra was set to 17,500 at* m*/*z* 200.

### 2.5. Analysis of Proteomic Data

Proteome Discoverer (version 1.3) software (Thermo Fisher Scientific) was used for protein identification and spectral count acquisition for each identified protein. The fragmentation spectra were searched against the* Brassica rapa* (Brassica V 1.2) protein database with precursor and fragment mass tolerances set to 10 ppm and 0.8 Da, respectively, and with up to two missed cleavages. Cysteine carbamidomethylation was set as a fixed modification, and methionine oxidation was set as a variable modification for database searching. Both peptide and protein identifications were filtered at 1% false discovery rate which were evaluated through decoy database which was created by reversing all of the* B. rapa* protein sequences. In addition the proteins identified with only one spectral count (SC) were discarded.

### 2.6. Comparative Analysis of Relative Protein Abundances

The Proteome Discoverer output was exported to Microsoft Excel to calculate normalized spectral counts (NSpC) [24–26]. The NSpC for each protein *k* is given by

(1) $$\begin{array}{c} {\left(\;NSpC\;\right)}_{k}=\frac{{\left(SpC/L\right)}_{k}}{\sum_{i=1}^{n}{\left(SpC/L\right)}_{i}}, \end{array}$$

where the total number of MS/MS spectra matching peptides from protein *k* (SpC) is divided by the protein's length (*L*) and then divided by SpC/*L* for all N proteins in the experiment.

### 2.7. Bioinformatics Analysis

Gene ontology (GO) annotations of* Brassica rapa* proteins were retrieved from BRAD* B. rapa* genome data V 1.2. GO enrichment analysis was performed in agriGO (http://bioinfo.cau.edu.cn/agriGO/) with customized parameters using the* B. rapa* whole genome as the background/reference. The clustering analyses were conducted with Genesis software [27] using centered correlation, and the average linkage procedure and tree were visualized with the same software. Composite expression profile analysis was performed by summing averages of NSpC for all proteins of a given functional category at each of the three time points of drought treatment.

## 3. Results and Discussion

### 3.1. Phenotypic Changes during Drought Stress

**Figure 1:**
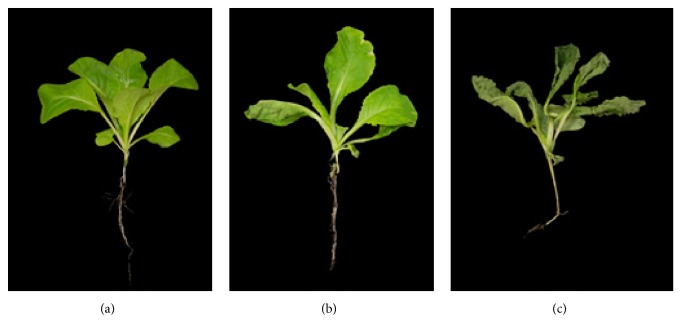
Response of* B. rapa* seedling during drought stress. (a) Control, (b) 24 h drought treatment, and (c) 48 h drought treatment.

*Brassica rapa* 3-week-old plants were subjected to drought stress for 0, 24, and 48 h (Figure 1). All leaves were withered on plants that were dehydrated for 24 h; the lower leaves were severely damaged so that few of the lower leaves were folded. Some leaves displayed slight chlorosis at the leaf margin. Similar effects were observed in plants subjected to dehydration for 48 h; all leaves were severely withered and folded, and slight chlorosis at the leaf margin was observed, similarly to that of plants dehydrated for 24 h. These morphological changes suggest that the drought treatment was effective, and the analyzed proteomes for plants subjected to 0 (control), 24, and 48 h of drought stress represent plant responses to normal conditions, mild drought stress, and severe drought stress, respectively.

### 3.2. Identification of Total Proteins from Young* Brassica rapa* Plants

**Figure 2:**
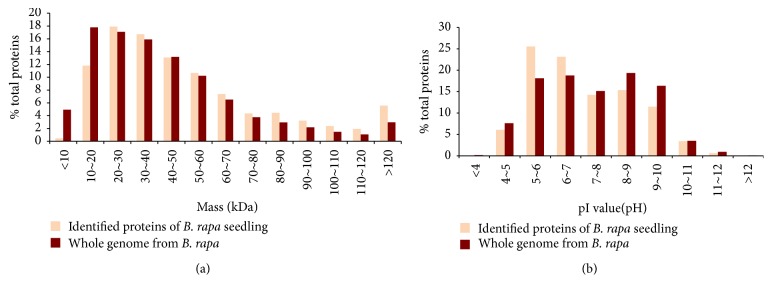
Distribution of molecular weight (a) and pI value (b) of proteins from* B. rapa* seedling (brown) and those of proteins encoded by the whole genome from* B. rapa* (orange).

Shotgun proteomic analysis was used to identify 3,009 nonredundant proteins (Supplementary Table S1 (see Supplementary Material available online at http://dx.doi.org/10.1155/2016/4235808)) with a false discovery rate of 0.01 after 0, 24, and 48 h of drought stress with three replicates. Shotgun proteomics analysis inherently contains a level of analytical incompleteness; the number of identified proteins for each sample ranged from 1,446 to 1,819 (Supplementary Table S2) [28]. The distributions of molecular weights (MWs) and pI values (Figure 2) for the identified* B. rapa* proteomes were compared with those of proteins encoded by the* Brassica* genome. The MWs of the identified proteins ranged from 5.5 kDa (Bra001019, unknown protein) to 534.5 kDa (Bra039167, the auxin transport protein BIG). The overall MW distribution of the identified proteins was similar to that of the* B. rapa* genome; however, the proportion of proteins less than 20 kDa was quite low and the proportion of large MW proteins was relatively high. This is possibly due to the fact that the low MW proteins contained a small number of amino acids and produced a small number of trypsin-digested peptides. Therefore, the chance that these small peptides were detected by mass spectrometry was relatively low. For the protein pI values, the lowest pH was 4.27 (Bra002368, calmodulin 5) and the highest pH was 12.2 (Bra035628, ribosomal protein S30 family protein) for identified proteins. Proteins in the range of pH 7 to pH 10 were slightly less abundant in the* B. rapa* genome. This is possibly due to technical difficulties in solubilizing basic proteins using our protein extraction method. However, more than 45.2% of the identified proteins were basic proteins (pI higher than pH 7), and the overall distribution of pI values of identified proteins was similar to that of the genomic proteins. This suggested that the* B. rapa* proteome identified by our shotgun proteomic analysis was not biased. The distribution of the identified proteins was similar to* B. rapa*. This unbiased protein identification indicated that shotgun proteomic analysis was suitable for evaluating global protein expression patterns in* B. rapa*.

We performed relative quantification of the identified* B. rapa* proteins using spectral count normalization. The most abundant proteins were ribulose-bisphosphate carboxylases (RuBisCOs; Bra028181, Bra028087, Bra028406, Bra025431, Bra041116, and Bra034028), chlorophyll a/b-binding proteins (Bra010807 and Bra030182), and light harvesting complex in photosystem II (Bra039070, Bra037913, Bra013183, Bra029732, Bra000708, Bra028906, Bra004989, and Bra026099). RuBisCO is involved in carbon fixation and is the most abundant protein in nature. Chlorophyll a/b-binding proteins have a role in light harvesting in the thylakoid in cooperation with the light harvesting complex in photosystem II [29, 30]. These three proteins have the highest expression in plant leaves. The relative amounts of RuBisCO, chlorophyll a/b-binding protein, and light harvesting complex in photosystem II were 9%, 2%, and 4% of the total protein. RuBisCO accounts for 30–50% of total plant protein in green tissues [31, 32]. The relative proportion of RuBisCO in our analyses was 9% and we detected the chlorophyll a/b-binding protein and the light harvesting complex in photosystem II accounted for 6% of total proteins. This possibly reflects the leaf tissues analyzed.

### 3.3. Identification of Differentially Expressed Proteins during Drought Stress

A total of 3,009 proteins were not reproducibly detected in all Gel-LC/MSMS analyses even among the replicates for the same treatment due to the phenomenon of analytical incompleteness. The randomly detected proteins can be misjudged as differentially expressed proteins; therefore, we excluded randomly detected proteins from comparative analysis. All proteins that were detected in all treatments and replicates were subjected to comparison analysis. For the remaining proteins that were not detected in all Gel-LC/MSMS runs, proteins were included in the comparison analysis if they were detected in all the three replicates for a certain treatment. Using this criterion, 1,567 proteins were subjected to comparative analysis. The relative protein quantity was estimated with SCs (refer to Section 2). The SCs for these 1,567 proteins were globally normalized (NSpC) followed by logarithmic transformation (natural log⁡(Ln) of NSpC) to conduct ANOVA (Supplementary Table S3). For the missing SC data, an arbitrary value of 0.1 was assigned to distinguish them with minimal SC of 2. The proteins identified with one SC were excluded in this study because the possible random identification even under 0.01 false positive cutoff. Thus the minimum difference of spectral count was 6 for the proteins were not detected one of the condition and judged as differentially expressed proteins. To evaluate the reproducibility of the 1,567 proteins, the coefficient of determination (*R* ^2^) between NSpCs for the biological replicates was estimated. The average *R* ^2^ was 0.93 and ranged from 0.88 to 0.95. This result suggested that the relative quantities of the 1,567 proteins were reproducible in the biological replicates. The comparative analysis detected 440 differentially expressed proteins (Supplementary Table S4) in 3-week-old plants during drought stress.

### 3.4. Clustering Analysis

**Figure 3:**
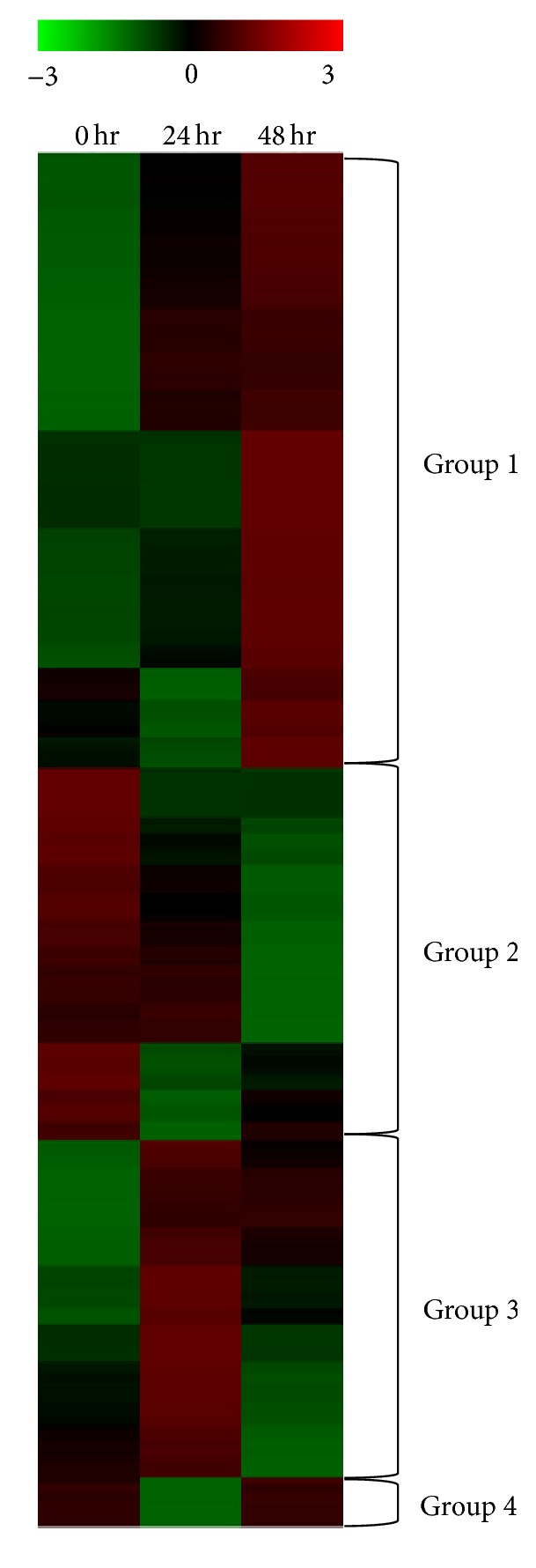
Clustering analysis for the 440 differentially expressed proteins.

The clustering analysis was conducted with 440 differentially expressed proteins, which were arbitrarily grouped into four groups based on similar expression patterns (Figure 3, Supplementary Table S5). Proteins in Group 1 increased steadily and showed highest expression at 48 h of drought treatment. A total of 197 proteins belonged to Group 1. The GO enrichment analysis of Group 1 proteins revealed that 85 GO terms (51 biological processes, 13 molecular functions, and 21 cellular components) (Supplementary Table S6) were enriched, suggesting that numerous metabolic changes occurred in response to drought stress. Many GO terms associated with catabolic processes were enriched. This may represent altered metabolism in drought-stressed plant cells, in which nutrients were not properly provided through photosynthesis, and catabolism was increased as a counteraction. Some proteins in this group were previously reported to be involved in resistance to various stresses. Twelve of the enriched GO terms were responses to various stresses, suggesting that these protein levels increased to respond to the drought stress, although their expression did not prevent leaf wilting at 48 h. Group 1 included proteins associated with autoxidation such as glutathione S-transferase and peroxidase, proteins associated with molecular chaperonin such as heat shock protein and late embryogenesis abundant proteins, and proteins associated with defense to biotic stress such as chitinase and PR proteins. Proteins previously reported as candidate genes for plant stress responses in other plant species also were detected in Group 1. Three annexins (Bra008737, Bra034402, and Bra036764) were detected. Annexins belong to a multigene family of Ca^2+^-dependent phospholipid- and cytoskeletal-binding proteins. Annexins are upregulated under various stress conditions. Overexpression of the mustard annexin (AnnBj1) in transgenic cotton plants conferred tolerance to various abiotic stresses such as sodium chloride, mannitol, polyethylene glycol, and hydrogen peroxide. In* Arabidopsis*, annexin had an important role in resistance to osmotic stress [33]. We observed that Bra008737 and Bra034402 levels increased rapidly during drought stress. A role for annexin has not been reported in* B. rapa*. The previously reported annexin functions associated with osmotic stress and drought tolerance in transgenic plants of other crop species suggest a possible role of annexin in drought stress tolerance in* B. rapa*. Group 1 also contains phospholipase D delta (Bra017730), which is involved in phospholipid metabolism responses to drought and salinity stress [34]. Cell membranes are major targets of environmental stresses, and lipids have crucial roles in preserving cell compartments under water stress conditions. In* Arabidopsis*, phospholipase D delta mRNA accumulated in response to dehydration and high salt stress [34, 35], suggesting a possible role of phospholipase D delta in drought tolerance through phospholipid metabolism.

The expression of 119 proteins in Group 2 steadily declined during drought treatment. GO enrichment analysis of Group 2 revealed enrichment of 90 GO terms (42 biological processes, 7 molecular functions, and 41 cellular components) (Supplementary Table S6). Among these terms, 34 were associated with photosynthesis, such as chloroplast organelle, photosynthesis dark and light reactions, and chlorophyll. The expression patterns of proteins in Group 2 primarily represent cell damage from drought stress. Drought stress reduces photosynthesis, and we observed that proteins associated with photosynthesis also were decreased. Proteins related to chlorophyll synthesis such as phytoene synthase (Bra006391), phytoene desaturase (Bra010751), and protochlorophyllide oxidoreductase (Bra026349) were downregulated during drought stress. Phytoene synthase catalysis generates two geranylgeranyl pyrophosphates. Phytoene synthase and protochlorophyllide oxidoreductase are related to carotenoid and ABA metabolism as well as chlorophyll [36–38]. Protochlorophyllide oxidoreductase catalyzes the phototransformation of protochlorophyllide to chlorophyllide [39]. Several ribosomal proteins were detected in Group 2, which also declined during drought stress. These reductions in proteins associated with photosynthesis and ribosomal proteins represent the cellular damage caused by drought stress. However, the auxin-responsive GH3 family protein (Bra006196) identified in Group 2 may be involved in resistance to drought stress, because it is involved in drought stress signaling pathways [40]. GH3 disrupts the conjugation of the phytohormone auxin (IAA) and amino acids by degrading free IAA and negatively regulating auxin homeostasis [41, 42]. Transgenic rice plants overexpressing GH3 were dwarf and had larger stomatal apertures, which were susceptible to water loss [43]. Thus, a reduction of GH3 in* B. rapa* may function to minimize water loss.

The 108 proteins in Group 3 had the highest expression levels at 24 h after the start of dehydration. The expression levels of some proteins in Group 3 were lower at 24 h compared to those at 0 h. However, the levels of all proteins in this group were enhanced by drought stress, and the expression levels of many proteins were higher at 42 h than at 0 h. Thus, the response of Group 3 is similar to that of Group 1, and the GO enrichment analysis was similar to that of Group 1. GO enrichment analysis of Group 3 revealed that 110 GO terms were enriched (69 biological processes, 16 molecular functions, and 25 cellular components) (Supplementary Table S6). GO terms associated with responses to various stresses and catabolic processes were enriched. Several antioxidant proteins were detected, including glutathione S-transferase F3 (Bra000311), peroxidase superfamily protein (Bra013576), stromal ascorbate peroxidase (Bra037859), manganese superoxide dismutase 1 (Bra029879), and copper/zinc superoxide dismutase 2 (Bra034394). These results for Groups 1 and 3 suggest that drought stress causes oxidative stress. Group 3 contains two candidate genes for resistance to drought stress. The level of ssDNA-binding transcriptional regulator (Bra024809) increased at 24 h. Gene expression modulation is an important cellular response to external stimuli, and transcriptional regulators have crucial roles in controlling transcription from specific genes in response to specific stimuli [44]. The target gene of ssDNA-binding transcriptional regulator is not known. However, the early increase in response to drought stress may suggest a role in activating gene(s) involved in drought resistance. Increased tumor necrosis factor receptor-associated factor- (TRAF-) like family protein (Bra020541) also was detected. TRAF-like family proteins are known to function in proteasome-mediated regulation during various developmental processes [45]. A knockout mutant in* Arabidopsis* of TRAF-like family protein had greater susceptibility to drought stress, and the TRAF-like family protein seven in absentia 2 (SINA2) promotes drought tolerance in an ABA-dependent manner in* Arabidopsis* [46].

The levels of 16 proteins in Group 4 were reduced at 24 h and recovered at 48 h. Only one GO term was enriched, the term for translation. Ribosomal proteins, translational initiation factor, and release factor are in Group 4. The reduced ribosomal protein levels at 24 h resulted from drought stress-mediated damage, similar to that observed in Group 2. However, the recovered expression levels at 48 h for proteins in Groups 2 and 4 were unexpected.

### 3.5. Composite Expression Profile of Functional Categories during Drought Treatment

**Figure 4:**
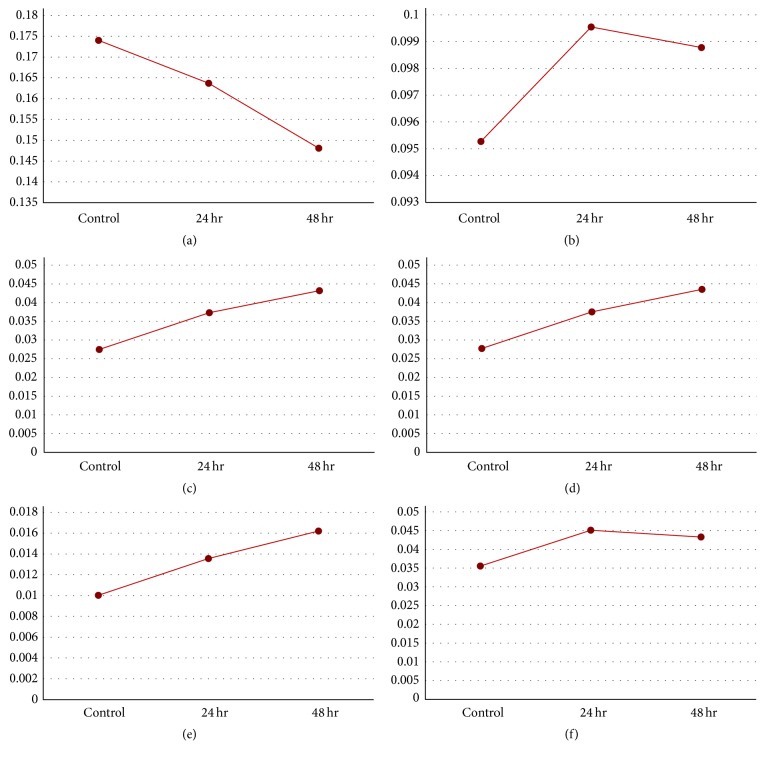
Composite protein expression patterns of gene function categories. (a) Chloroplast, (b) response to abiotic stimulus, (c) response to salt stress, (d) response to osmotic stress, (e) antioxidant activity, and (f) catabolic process.

To characterize global protein expression patterns involved in specific processes, expression graphs were represented by summing NSpC for each protein in each functional category according to the functional catalog by Bevan et al. [47] (Figure 4). The results of GO enrichment analysis for each clustered group indicate that functional categories of chloroplast, catabolic process, response to abiotic stimulus, response to osmotic stress, and antioxidant activity were representative of the molecular changes that occurred during drought stress in* B. rapa*. The composite expression profiles of those five representative functional GO categories for all 440 differentially expressed proteins were analyzed. The results of these composite analyses were consistent with the results of the GO enrichment analysis for the individual groups. The composite expression patterns of proteins in chloroplast categories continuously declined, whereas those of the catabolic process category continuously increased. The responses of these two functional categories revealed the responses to drought stress-mediated cellular damage. For the response to abiotic stimulus, protein expression rapidly increased and remained high in response to drought stress treatment, although protein expression slightly declined at 48 h compared with that at 24 h. Proteins involved in response to osmotic stress and antioxidant activity were continuously expressed during drought stress. The temporal expression patterns of these proteins suggest that they could be evaluated as candidate proteins for drought tolerance.

## 4. Conclusion

We performed shotgun proteomic analysis and identified 3,009 nonredundant proteins in young* B. rapa* plants. RuBisCOs, chlorophyll a/b-binding protein, and light harvesting complex in photosystem II were abundantly expressed in* B. rapa* leaf tissues.

We compared the relative abundance of 1,567 reproducibly detected proteins and identified 440 proteins that were differentially expressed in response to drought stress. Our quantitative proteomics study using clustering analysis, GO enrichment analysis, and composite expression profiles of functional categories with the 440 differentially expressed proteins provides comprehensive molecular insights into protein level changes and modifications induced by drought stress. The representative responses to drought stress included a reduction in proteins associated with photosynthesis and an increase in proteins responding to various stresses. The expression of proteins involved in antioxidant activities increased during drought stress. These proteins likely have important roles in the removal of active oxygen species produced during drought stress. The* B. rapa* inbred line “Chiifu” is not drought tolerant and did not show a drought resistant morphology during 48 h of the experiment. However, we detected the induction of many proteins involved in abiotic stress responses, including osmotic stress, and proteins involved in antioxidant reactions. We propose that annexin, phospholipase D delta, ssDNA-binding transcriptional regulator, auxin-responsive GH3 family protein, and TRAF-like family protein are candidate genes for engineering drought tolerance or drought resistance in* B. rapa* based on their expression patterns under drought stress and previously reported molecular functions.

## Supplementary Material

Supplementary table 1. List of total 3,009 identified non-redundant proteins.
Supplementary table 2. List of identified non-redundant proteins from each sample.
Supplementary table 3. Relative expression amount of the 1,567 proteins among the treatments and replications.
Supplementary table 4. Relative amount of 440 differentially expressed proteins.
Supplementary table 5. Average of relative amount of proteins in each groups.
Supplementary table 6. Enriched GO terms of proteins in each groups.
