# Supplementary material for: Shotgun Quantitative Proteomic Analysis of Proteins Responding to Drought Stress in Brassica rapa L. (Inbred Line “Chiifu”)
Source: Int J Genomics. 2016 Jun 21;2016:4235808. doi: 10.1155/2016/4235808 (PMC4932182; doi:10.1155/2016/4235808)
Supplement: Supplementary file 1 — Supplementary table 1. List of total 3,009 identified non-redundant proteins. Supplementary table 2. List of identified non-redundant proteins from each sample. Supplementary table 3. Relative expression amount of the 1,567 proteins among the treatments and replications. Supplementary table 4. Relative amount of 440 differentially expressed proteins. Supplementary table 5. Average of relative amount of proteins in each groups. Supplementary table 6. Enriched GO terms of proteins in each groups. [file 4235808.f1.zip › sup.table S6.docx]

Supplementary table 6. Enriched GO terms of proteins in each groups

|  | GO term | Ontology^a)^ | Description | Number in input list | Number in BG/Ref | p-value | FDR |
| --- | --- | --- | --- | --- | --- | --- | --- |
| group1 | GO:0010038 | P | response to metal ion | 31 | 725 | 7.70E-16 | 7.10E-13 |
|  | GO:0010035 | P | response to inorganic substance | 34 | 976 | 1.30E-14 | 6.20E-12 |
|  | GO:0046686 | P | response to cadmium ion | 26 | 585 | 5.10E-14 | 1.60E-11 |
|  | GO:0006970 | P | response to osmotic stress | 25 | 734 | 4.20E-11 | 9.70E-09 |
|  | GO:0009651 | P | response to salt stress | 24 | 685 | 5.60E-11 | 1.00E-08 |
|  | GO:0044282 | P | small molecule catabolic process | 19 | 431 | 1.10E-10 | 1.60E-08 |
|  | GO:0044248 | P | cellular catabolic process | 31 | 1442 | 1.60E-08 | 2.10E-06 |
|  | GO:0006006 | P | glucose metabolic process | 13 | 262 | 1.90E-08 | 2.20E-06 |
|  | GO:0009056 | P | catabolic process | 36 | 1902 | 3.30E-08 | 3.30E-06 |
|  | GO:0016054 | P | organic acid catabolic process | 10 | 154 | 6.40E-08 | 5.40E-06 |
|  | GO:0046395 | P | carboxylic acid catabolic process | 10 | 154 | 6.40E-08 | 5.40E-06 |
|  | GO:0009063 | P | cellular amino acid catabolic process | 8 | 100 | 2.60E-07 | 1.90E-05 |
|  | GO:0019318 | P | hexose metabolic process | 13 | 329 | 2.70E-07 | 1.90E-05 |
|  | GO:0009310 | P | amine catabolic process | 8 | 104 | 3.50E-07 | 2.30E-05 |
|  | GO:0009064 | P | glutamine family amino acid metabolic process | 9 | 160 | 9.60E-07 | 5.90E-05 |
|  | GO:0006536 | P | glutamate metabolic process | 6 | 55 | 1.30E-06 | 7.50E-05 |
|  | GO:0005996 | P | monosaccharide metabolic process | 13 | 386 | 1.60E-06 | 8.50E-05 |
|  | GO:0043436 | P | oxoacid metabolic process | 28 | 1550 | 1.90E-06 | 9.20E-05 |
|  | GO:0019752 | P | carboxylic acid metabolic process | 28 | 1550 | 1.90E-06 | 9.20E-05 |
|  | GO:0006082 | P | organic acid metabolic process | 28 | 1557 | 2.10E-06 | 9.50E-05 |
|  | GO:0016052 | P | carbohydrate catabolic process | 13 | 402 | 2.40E-06 | 0.0001 |
|  | GO:0044281 | P | small molecule metabolic process | 45 | 3184 | 2.50E-06 | 0.0001 |
|  | GO:0042180 | P | cellular ketone metabolic process | 28 | 1575 | 2.50E-06 | 0.0001 |
|  | GO:0042221 | P | response to chemical stimulus | 53 | 4019 | 3.00E-06 | 0.00012 |
|  | GO:0009628 | P | response to abiotic stimulus | 34 | 2193 | 4.90E-06 | 0.00018 |
|  | GO:0019320 | P | hexose catabolic process | 9 | 204 | 7.00E-06 | 0.00023 |
|  | GO:0046365 | P | monosaccharide catabolic process | 9 | 204 | 7.00E-06 | 0.00023 |
|  | GO:0006007 | P | glucose catabolic process | 9 | 203 | 6.70E-06 | 0.00023 |
|  | GO:0006096 | P | glycolysis | 8 | 155 | 7.10E-06 | 0.00023 |
|  | GO:0009409 | P | response to cold | 13 | 449 | 7.90E-06 | 0.00024 |
|  | GO:0046164 | P | alcohol catabolic process | 9 | 225 | 1.50E-05 | 0.00045 |
|  | GO:0044275 | P | cellular carbohydrate catabolic process | 10 | 287 | 1.70E-05 | 0.0005 |
|  | GO:0034641 | P | cellular nitrogen compound metabolic process | 21 | 1139 | 2.20E-05 | 0.00061 |
|  | GO:0006950 | P | response to stress | 53 | 4360 | 2.30E-05 | 0.00063 |
|  | GO:0055114 | P | oxidation reduction | 34 | 2378 | 2.40E-05 | 0.00063 |
|  | GO:0044262 | P | cellular carbohydrate metabolic process | 20 | 1067 | 2.60E-05 | 0.00068 |
|  | GO:0009266 | P | response to temperature stimulus | 15 | 682 | 4.20E-05 | 0.001 |
|  | GO:0006091 | P | generation of precursor metabolites and energy | 16 | 769 | 4.60E-05 | 0.0011 |
|  | GO:0009308 | P | amine metabolic process | 18 | 940 | 4.80E-05 | 0.0011 |
|  | GO:0044106 | P | cellular amine metabolic process | 16 | 789 | 6.10E-05 | 0.0014 |
|  | GO:0006066 | P | alcohol metabolic process | 14 | 632 | 6.70E-05 | 0.0015 |
|  | GO:0006520 | P | cellular amino acid metabolic process | 15 | 715 | 7.00E-05 | 0.0015 |
|  | GO:0006508 | P | proteolysis | 19 | 1081 | 9.20E-05 | 0.002 |
|  | GO:0050896 | P | response to stimulus | 75 | 7170 | 0.0001 | 0.0021 |
|  | GO:0006979 | P | response to oxidative stress | 12 | 519 | 0.00014 | 0.0029 |
|  | GO:0005975 | P | carbohydrate metabolic process | 25 | 1750 | 0.00022 | 0.0045 |
|  | GO:0043648 | P | dicarboxylic acid metabolic process | 6 | 139 | 0.00026 | 0.005 |
|  | GO:0009057 | P | macromolecule catabolic process | 17 | 1002 | 0.0003 | 0.0057 |
|  | GO:0006519 | P | cellular amino acid and derivative metabolic process | 20 | 1326 | 0.00043 | 0.0081 |
|  | GO:0009820 | P | alkaloid metabolic process | 6 | 162 | 0.00058 | 0.011 |
|  | GO:0044265 | P | cellular macromolecule catabolic process | 13 | 825 | 0.0026 | 0.046 |
|  | GO:0005507 | F | copper ion binding | 14 | 376 | 2.00E-07 | 7.50E-05 |
|  | GO:0048037 | F | cofactor binding | 20 | 861 | 1.20E-06 | 0.00024 |
|  | GO:0016903 | F | oxidoreductase activity, acting on the aldehyde or oxo group of donors | 7 | 105 | 4.90E-06 | 0.00047 |
|  | GO:0016491 | F | oxidoreductase activity | 36 | 2385 | 4.90E-06 | 0.00047 |
|  | GO:0004175 | F | endopeptidase activity | 14 | 517 | 7.80E-06 | 0.00059 |
|  | GO:0070011 | F | peptidase activity, acting on L-amino acid peptides | 18 | 896 | 2.60E-05 | 0.0016 |
|  | GO:0008483 | F | transaminase activity | 6 | 104 | 5.20E-05 | 0.0022 |
|  | GO:0008233 | F | peptidase activity | 18 | 945 | 5.10E-05 | 0.0022 |
|  | GO:0050662 | F | coenzyme binding | 14 | 604 | 4.20E-05 | 0.0022 |
|  | GO:0016620 | F | oxidoreductase activity, acting on the aldehyde or oxo group of donors, NAD or NADP as acceptor | 5 | 73 | 9.70E-05 | 0.0037 |
|  | GO:0016769 | F | transferase activity, transferring nitrogenous groups | 6 | 127 | 0.00016 | 0.0054 |
|  | GO:0003824 | F | catalytic activity | 128 | 14101 | 0.00042 | 0.013 |
|  | GO:0016209 | F | antioxidant activity | 7 | 230 | 0.00067 | 0.019 |
|  | GO:0005773 | C | vacuole | 32 | 1299 | 4.20E-10 | 1.30E-07 |
|  | GO:0009536 | C | plastid | 46 | 2854 | 6.90E-08 | 1.10E-05 |
|  | GO:0009507 | C | chloroplast | 45 | 2811 | 1.10E-07 | 1.10E-05 |
|  | GO:0005739 | C | mitochondrion | 34 | 1874 | 1.80E-07 | 1.40E-05 |
|  | GO:0044444 | C | cytoplasmic part | 107 | 9427 | 5.20E-07 | 3.30E-05 |
|  | GO:0048046 | C | apoplast | 17 | 618 | 7.70E-07 | 4.00E-05 |
|  | GO:0005737 | C | cytoplasm | 126 | 11818 | 1.60E-06 | 7.10E-05 |
|  | GO:0005777 | C | peroxisome | 10 | 267 | 9.30E-06 | 0.00033 |
|  | GO:0042579 | C | microbody | 10 | 267 | 9.30E-06 | 0.00033 |
|  | GO:0005618 | C | cell wall | 22 | 1190 | 1.40E-05 | 0.00044 |
|  | GO:0005741 | C | mitochondrial outer membrane | 6 | 85 | 1.70E-05 | 0.00048 |
|  | GO:0030312 | C | external encapsulating structure | 22 | 1231 | 2.30E-05 | 0.0006 |
|  | GO:0044429 | C | mitochondrial part | 15 | 683 | 4.20E-05 | 0.001 |
|  | GO:0031968 | C | organelle outer membrane | 6 | 132 | 0.00019 | 0.0044 |
|  | GO:0019867 | C | outer membrane | 6 | 138 | 0.00025 | 0.0052 |
|  | GO:0005886 | C | plasma membrane | 48 | 4409 | 0.00048 | 0.0084 |
|  | GO:0005759 | C | mitochondrial matrix | 6 | 155 | 0.00046 | 0.0084 |
|  | GO:0031980 | C | mitochondrial lumen | 6 | 155 | 0.00046 | 0.0084 |
|  | GO:0005829 | C | cytosol | 21 | 1449 | 0.00053 | 0.0087 |
|  | GO:0005740 | C | mitochondrial envelope | 11 | 537 | 0.0007 | 0.011 |
|  | GO:0009579 | C | thylakoid | 11 | 572 | 0.0012 | 0.017 |
| group2 | GO:0015979 | P | photosynthesis | 17 | 258 | 9.40E-16 | 4.60E-13 |
|  | GO:0019685 | P | photosynthesis, dark reaction | 6 | 39 | 8.70E-09 | 1.10E-06 |
|  | GO:0044271 | P | cellular nitrogen compound biosynthetic process | 16 | 609 | 4.80E-09 | 1.10E-06 |
|  | GO:0019253 | P | reductive pentose-phosphate cycle | 6 | 39 | 8.70E-09 | 1.10E-06 |
|  | GO:0015977 | P | carbon fixation | 6 | 47 | 2.80E-08 | 2.30E-06 |
|  | GO:0071704 | P | organic substance metabolic process | 6 | 47 | 2.80E-08 | 2.30E-06 |
|  | GO:0042742 | P | defense response to bacterium | 13 | 448 | 3.70E-08 | 2.50E-06 |
|  | GO:0015995 | P | chlorophyll biosynthetic process | 7 | 84 | 4.00E-08 | 2.50E-06 |
|  | GO:0043094 | P | cellular metabolic compound salvage | 7 | 93 | 8.20E-08 | 4.50E-06 |
|  | GO:0006779 | P | porphyrin biosynthetic process | 7 | 100 | 1.30E-07 | 6.10E-06 |
|  | GO:0046148 | P | pigment biosynthetic process | 9 | 204 | 1.30E-07 | 6.10E-06 |
|  | GO:0033014 | P | tetrapyrrole biosynthetic process | 7 | 104 | 1.80E-07 | 6.70E-06 |
|  | GO:0009853 | P | photorespiration | 6 | 63 | 1.70E-07 | 6.70E-06 |
|  | GO:0009617 | P | response to bacterium | 13 | 534 | 2.70E-07 | 8.40E-06 |
|  | GO:0015994 | P | chlorophyll metabolic process | 7 | 109 | 2.40E-07 | 8.40E-06 |
|  | GO:0034641 | P | cellular nitrogen compound metabolic process | 19 | 1139 | 2.60E-07 | 8.40E-06 |
|  | GO:0042440 | P | pigment metabolic process | 9 | 237 | 4.50E-07 | 1.30E-05 |
|  | GO:0006778 | P | porphyrin metabolic process | 7 | 129 | 7.60E-07 | 2.10E-05 |
|  | GO:0033013 | P | tetrapyrrole metabolic process | 7 | 131 | 8.50E-07 | 2.20E-05 |
|  | GO:0055114 | P | oxidation reduction | 26 | 2378 | 6.10E-06 | 1.50E-04 |
|  | GO:0018130 | P | heterocycle biosynthetic process | 8 | 250 | 6.60E-06 | 1.60E-04 |
|  | GO:0009409 | P | response to cold | 10 | 449 | 1.20E-05 | 2.80E-04 |
|  | GO:0051707 | P | response to other organism | 15 | 1036 | 2.00E-05 | 0.00042 |
|  | GO:0016051 | P | carbohydrate biosynthetic process | 10 | 485 | 2.40E-05 | 0.00048 |
|  | GO:0008652 | P | cellular amino acid biosynthetic process | 8 | 319 | 3.80E-05 | 0.00074 |
|  | GO:0019684 | P | photosynthesis, light reaction | 5 | 102 | 4.70E-05 | 0.00089 |
|  | GO:0046483 | P | heterocycle metabolic process | 12 | 788 | 7.40E-05 | 0.0013 |
|  | GO:0051188 | P | cofactor biosynthetic process | 7 | 261 | 7.40E-05 | 0.0013 |
|  | GO:0009607 | P | response to biotic stimulus | 15 | 1188 | 8.70E-05 | 0.0014 |
|  | GO:0009266 | P | response to temperature stimulus | 11 | 682 | 8.60E-05 | 0.0014 |
|  | GO:0009309 | P | amine biosynthetic process | 8 | 366 | 9.70E-05 | 0.0015 |
|  | GO:0006520 | P | cellular amino acid metabolic process | 11 | 715 | 0.00013 | 0.002 |
|  | GO:0006091 | P | generation of precursor metabolites and energy | 11 | 769 | 0.00024 | 0.0036 |
|  | GO:0051704 | P | multi-organism process | 16 | 1464 | 0.00026 | 0.0038 |
|  | GO:0044106 | P | cellular amine metabolic process | 11 | 789 | 0.0003 | 0.0042 |
|  | GO:0022900 | P | electron transport chain | 7 | 356 | 0.00048 | 0.0066 |
|  | GO:0006952 | P | defense response | 16 | 1611 | 0.0007 | 0.0094 |
|  | GO:0006412 | P | translation | 12 | 1079 | 0.0011 | 0.015 |
|  | GO:0009308 | P | amine metabolic process | 11 | 940 | 0.0012 | 0.015 |
|  | GO:0016053 | P | organic acid biosynthetic process | 10 | 809 | 0.0013 | 0.016 |
|  | GO:0046394 | P | carboxylic acid biosynthetic process | 10 | 809 | 0.0013 | 0.016 |
|  | GO:0051186 | P | cofactor metabolic process | 7 | 468 | 0.0023 | 0.027 |
|  | GO:0016829 | F | lyase activity | 11 | 580 | 2.10E-05 | 0.0036 |
|  | GO:0048037 | F | cofactor binding | 13 | 861 | 4.20E-05 | 0.0037 |
|  | GO:0050662 | F | coenzyme binding | 10 | 604 | 0.00014 | 0.0083 |
|  | GO:0016491 | F | oxidoreductase activity | 22 | 2385 | 0.00026 | 0.012 |
|  | GO:0016830 | F | carbon-carbon lyase activity | 5 | 159 | 0.00037 | 0.013 |
|  | GO:0051536 | F | iron-sulfur cluster binding | 5 | 181 | 0.00067 | 0.017 |
|  | GO:0051540 | F | metal cluster binding | 5 | 181 | 6.70E-04 | 0.017 |
|  | GO:0044435 | C | plastid part | 50 | 1324 | 9.90E-30 | 1.40E-27 |
|  | GO:0044434 | C | chloroplast part | 50 | 1306 | 5.40E-30 | 1.40E-27 |
|  | GO:0009507 | C | chloroplast | 69 | 2811 | 3.80E-28 | 3.70E-26 |
|  | GO:0009536 | C | plastid | 69 | 2854 | 8.70E-28 | 6.40E-26 |
|  | GO:0009532 | C | plastid stroma | 32 | 618 | 2.30E-24 | 1.30E-22 |
|  | GO:0009570 | C | chloroplast stroma | 31 | 599 | 1.10E-23 | 5.20E-22 |
|  | GO:0009579 | C | thylakoid | 28 | 572 | 4.80E-21 | 2.00E-19 |
|  | GO:0009526 | C | plastid envelope | 29 | 661 | 2.00E-20 | 7.40E-19 |
|  | GO:0009941 | C | chloroplast envelope | 28 | 645 | 1.10E-19 | 3.50E-18 |
|  | GO:0055035 | C | plastid thylakoid membrane | 23 | 421 | 9.10E-19 | 2.40E-17 |
|  | GO:0009535 | C | chloroplast thylakoid membrane | 23 | 421 | 9.10E-19 | 2.40E-17 |
|  | GO:0042651 | C | thylakoid membrane | 23 | 438 | 2.20E-18 | 5.30E-17 |
|  | GO:0034357 | C | photosynthetic membrane | 23 | 449 | 3.70E-18 | 8.30E-17 |
|  | GO:0031976 | C | plastid thylakoid | 23 | 467 | 8.70E-18 | 1.60E-16 |
|  | GO:0044436 | C | thylakoid part | 23 | 466 | 8.30E-18 | 1.60E-16 |
|  | GO:0009534 | C | chloroplast thylakoid | 23 | 467 | 8.70E-18 | 1.60E-16 |
|  | GO:0031984 | C | organelle subcompartment | 23 | 543 | 2.20E-16 | 3.80E-15 |
|  | GO:0031967 | C | organelle envelope | 32 | 1316 | 4.90E-15 | 8.00E-14 |
|  | GO:0031975 | C | envelope | 32 | 1356 | 1.10E-14 | 1.70E-13 |
|  | GO:0048046 | C | apoplast | 20 | 618 | 2.10E-12 | 3.00E-11 |
|  | GO:0010319 | C | stromule | 8 | 59 | 8.50E-11 | 1.20E-09 |
|  | GO:0010287 | C | plastoglobule | 9 | 93 | 1.30E-10 | 1.70E-09 |
|  | GO:0044444 | C | cytoplasmic part | 83 | 9427 | 4.30E-09 | 5.40E-08 |
|  | GO:0044446 | C | intracellular organelle part | 60 | 6476 | 3.70E-08 | 4.50E-07 |
|  | GO:0044422 | C | organelle part | 60 | 6488 | 3.90E-08 | 4.50E-07 |
|  | GO:0005737 | C | cytoplasm | 90 | 11818 | 3.50E-07 | 3.90E-06 |
|  | GO:0031969 | C | chloroplast membrane | 7 | 205 | 1.60E-05 | 1.70E-04 |
|  | GO:0042170 | C | plastid membrane | 7 | 211 | 1.90E-05 | 2.00E-04 |
|  | GO:0005576 | C | extracellular region | 22 | 2029 | 2.90E-05 | 3.00E-04 |
|  | GO:0031090 | C | organelle membrane | 29 | 3165 | 4.60E-05 | 4.40E-04 |
|  | GO:0009521 | C | photosystem | 5 | 113 | 7.60E-05 | 7.20E-04 |
|  | GO:0032991 | C | macromolecular complex | 30 | 3542 | 1.30E-04 | 0.0012 |
|  | GO:0030529 | C | ribonucleoprotein complex | 14 | 1217 | 0.00036 | 0.0032 |
|  | GO:0005840 | C | ribosome | 11 | 814 | 0.00038 | 0.0033 |
|  | GO:0005739 | C | mitochondrion | 16 | 1874 | 0.003 | 0.025 |
|  | GO:0043229 | C | intracellular organelle | 83 | 15219 | 0.0044 | 0.035 |
|  | GO:0043226 | C | organelle | 83 | 15228 | 0.0044 | 0.035 |
|  | GO:0043231 | C | intracellular membrane-bounded organelle | 79 | 14541 | 0.0053 | 0.041 |
|  | GO:0043227 | C | membrane-bounded organelle | 79 | 14558 | 0.0055 | 0.041 |
|  | GO:0044424 | C | intracellular part | 92 | 17299 | 0.0056 | 0.041 |
|  | GO:0005622 | C | intracellular | 93 | 17663 | 0.0067 | 0.048 |
| group3 | GO:0010038 | P | response to metal ion | 22 | 725 | 1.50E-13 | 6.20E-11 |
|  | GO:0010035 | P | response to inorganic substance | 25 | 976 | 1.80E-13 | 6.20E-11 |
|  | GO:0008652 | P | cellular amino acid biosynthetic process | 15 | 319 | 1.70E-12 | 3.00E-10 |
|  | GO:0046686 | P | response to cadmium ion | 19 | 585 | 1.70E-12 | 3.00E-10 |
|  | GO:0044281 | P | small molecule metabolic process | 44 | 3184 | 2.40E-12 | 3.30E-10 |
|  | GO:0009309 | P | amine biosynthetic process | 15 | 366 | 1.20E-11 | 1.40E-09 |
|  | GO:0044271 | P | cellular nitrogen compound biosynthetic process | 18 | 609 | 2.80E-11 | 2.40E-09 |
|  | GO:0044248 | P | cellular catabolic process | 27 | 1442 | 2.60E-11 | 2.40E-09 |
|  | GO:0009056 | P | catabolic process | 31 | 1902 | 3.60E-11 | 2.80E-09 |
|  | GO:0043436 | P | oxoacid metabolic process | 27 | 1550 | 1.20E-10 | 7.60E-09 |
|  | GO:0019752 | P | carboxylic acid metabolic process | 27 | 1550 | 1.20E-10 | 7.60E-09 |
|  | GO:0006082 | P | organic acid metabolic process | 27 | 1557 | 1.30E-10 | 7.70E-09 |
|  | GO:0042180 | P | cellular ketone metabolic process | 27 | 1575 | 1.70E-10 | 9.00E-09 |
|  | GO:0006006 | P | glucose metabolic process | 12 | 262 | 3.20E-10 | 1.60E-08 |
|  | GO:0034641 | P | cellular nitrogen compound metabolic process | 22 | 1139 | 7.10E-10 | 3.30E-08 |
|  | GO:0044275 | P | cellular carbohydrate catabolic process | 12 | 287 | 9.00E-10 | 3.90E-08 |
|  | GO:0016053 | P | organic acid biosynthetic process | 18 | 809 | 2.30E-09 | 9.00E-08 |
|  | GO:0046394 | P | carboxylic acid biosynthetic process | 18 | 809 | 2.30E-09 | 9.00E-08 |
|  | GO:0010043 | P | response to zinc ion | 7 | 64 | 3.40E-09 | 1.20E-07 |
|  | GO:0016052 | P | carbohydrate catabolic process | 13 | 402 | 4.20E-09 | 1.30E-07 |
|  | GO:0019320 | P | hexose catabolic process | 10 | 204 | 4.60E-09 | 1.30E-07 |
|  | GO:0019318 | P | hexose metabolic process | 12 | 329 | 4.10E-09 | 1.30E-07 |
|  | GO:0006007 | P | glucose catabolic process | 10 | 203 | 4.40E-09 | 1.30E-07 |
|  | GO:0046365 | P | monosaccharide catabolic process | 10 | 204 | 4.60E-09 | 1.30E-07 |
|  | GO:0044282 | P | small molecule catabolic process | 13 | 431 | 9.40E-09 | 2.60E-07 |
|  | GO:0044106 | P | cellular amine metabolic process | 17 | 789 | 9.70E-09 | 2.60E-07 |
|  | GO:0046164 | P | alcohol catabolic process | 10 | 225 | 1.20E-08 | 3.00E-07 |
|  | GO:0000097 | P | sulfur amino acid biosynthetic process | 7 | 78 | 1.40E-08 | 3.40E-07 |
|  | GO:0006520 | P | cellular amino acid metabolic process | 16 | 715 | 1.50E-08 | 3.50E-07 |
|  | GO:0006066 | P | alcohol metabolic process | 15 | 632 | 1.80E-08 | 4.30E-07 |
|  | GO:0009308 | P | amine metabolic process | 18 | 940 | 2.20E-08 | 5.00E-07 |
|  | GO:0005996 | P | monosaccharide metabolic process | 12 | 386 | 2.40E-08 | 5.20E-07 |
|  | GO:0009057 | P | macromolecule catabolic process | 18 | 1002 | 5.60E-08 | 1.20E-06 |
|  | GO:0044265 | P | cellular macromolecule catabolic process | 16 | 825 | 1.00E-07 | 2.00E-06 |
|  | GO:0006096 | P | glycolysis | 8 | 155 | 1.00E-07 | 2.00E-06 |
|  | GO:0044262 | P | cellular carbohydrate metabolic process | 18 | 1067 | 1.40E-07 | 2.70E-06 |
|  | GO:0000096 | P | sulfur amino acid metabolic process | 7 | 132 | 5.20E-07 | 9.70E-06 |
|  | GO:0043648 | P | dicarboxylic acid metabolic process | 7 | 139 | 7.30E-07 | 1.30E-05 |
|  | GO:0044283 | P | small molecule biosynthetic process | 20 | 1479 | 9.50E-07 | 1.70E-05 |
|  | GO:0006091 | P | generation of precursor metabolites and energy | 14 | 769 | 1.20E-06 | 2.00E-05 |
|  | GO:0006732 | P | coenzyme metabolic process | 9 | 302 | 1.70E-06 | 2.90E-05 |
|  | GO:0005975 | P | carbohydrate metabolic process | 21 | 1750 | 3.30E-06 | 5.40E-05 |
|  | GO:0006099 | P | tricarboxylic acid cycle | 5 | 77 | 8.10E-06 | 0.00013 |
|  | GO:0046356 | P | acetyl-CoA catabolic process | 5 | 77 | 8.10E-06 | 0.00013 |
|  | GO:0051186 | P | cofactor metabolic process | 10 | 468 | 8.80E-06 | 0.00014 |
|  | GO:0006519 | P | cellular amino acid and derivative metabolic process | 17 | 1326 | 1.00E-05 | 0.00016 |
|  | GO:0009109 | P | coenzyme catabolic process | 5 | 82 | 1.10E-05 | 0.00016 |
|  | GO:0044272 | P | sulfur compound biosynthetic process | 7 | 213 | 1.20E-05 | 0.00018 |
|  | GO:0006084 | P | acetyl-CoA metabolic process | 5 | 85 | 1.30E-05 | 0.00019 |
|  | GO:0009060 | P | aerobic respiration | 5 | 88 | 1.50E-05 | 0.00022 |
|  | GO:0015980 | P | energy derivation by oxidation of organic compounds | 6 | 163 | 2.70E-05 | 0.00036 |
|  | GO:0006790 | P | sulfur metabolic process | 8 | 337 | 3.10E-05 | 0.00042 |
|  | GO:0055114 | P | oxidation reduction | 23 | 2378 | 3.40E-05 | 0.00044 |
|  | GO:0046483 | P | heterocycle metabolic process | 12 | 788 | 3.50E-05 | 0.00045 |
|  | GO:0051187 | P | cofactor catabolic process | 5 | 107 | 4.00E-05 | 0.0005 |
|  | GO:0042221 | P | response to chemical stimulus | 32 | 4019 | 6.40E-05 | 0.00079 |
|  | GO:0055086 | P | nucleobase, nucleoside and nucleotide metabolic process | 9 | 487 | 7.10E-05 | 0.00087 |
|  | GO:0045333 | P | cellular respiration | 5 | 124 | 8.00E-05 | 0.00096 |
|  | GO:0006800 | P | oxygen and reactive oxygen species metabolic process | 6 | 216 | 0.00012 | 0.0015 |
|  | GO:0006730 | P | one-carbon metabolic process | 5 | 199 | 0.00071 | 0.0082 |
|  | GO:0009117 | P | nucleotide metabolic process | 7 | 437 | 0.00098 | 0.011 |
|  | GO:0006753 | P | nucleoside phosphate metabolic process | 7 | 437 | 0.00098 | 0.011 |
|  | GO:0005976 | P | polysaccharide metabolic process | 7 | 465 | 0.0014 | 0.015 |
|  | GO:0018130 | P | heterocycle biosynthetic process | 5 | 250 | 0.0019 | 0.021 |
|  | GO:0032787 | P | monocarboxylic acid metabolic process | 9 | 787 | 0.0021 | 0.023 |
|  | GO:0006163 | P | purine nucleotide metabolic process | 5 | 266 | 0.0025 | 0.026 |
|  | GO:0006979 | P | response to oxidative stress | 7 | 519 | 0.0025 | 0.026 |
|  | GO:0009651 | P | response to salt stress | 8 | 685 | 0.0032 | 0.032 |
|  | GO:0006970 | P | response to osmotic stress | 8 | 734 | 0.0047 | 0.048 |
|  | GO:0019842 | F | vitamin binding | 9 | 344 | 4.80E-06 | 0.00046 |
|  | GO:0048037 | F | cofactor binding | 14 | 861 | 4.20E-06 | 0.00046 |
|  | GO:0016616 | F | oxidoreductase activity, acting on the CH-OH group of donors, NAD or NADP as acceptor | 8 | 281 | 8.60E-06 | 0.00055 |
|  | GO:0016209 | F | antioxidant activity | 7 | 230 | 2.00E-05 | 0.00096 |
|  | GO:0016614 | F | oxidoreductase activity, acting on CH-OH group of donors | 8 | 339 | 3.20E-05 | 0.0011 |
|  | GO:0016491 | F | oxidoreductase activity | 23 | 2385 | 3.50E-05 | 0.0011 |
|  | GO:0016684 | F | oxidoreductase activity, acting on peroxide as acceptor | 5 | 190 | 0.00057 | 0.011 |
|  | GO:0004601 | F | peroxidase activity | 5 | 190 | 0.00057 | 0.011 |
|  | GO:0005507 | F | copper ion binding | 7 | 376 | 0.00041 | 0.011 |
|  | GO:0016853 | F | isomerase activity | 7 | 387 | 0.00048 | 0.011 |
|  | GO:0030170 | F | pyridoxal phosphate binding | 5 | 210 | 0.0009 | 0.014 |
|  | GO:0070279 | F | vitamin B6 binding | 5 | 210 | 0.0009 | 0.014 |
|  | GO:0016829 | F | lyase activity | 8 | 580 | 0.0012 | 0.016 |
|  | GO:0000287 | F | magnesium ion binding | 5 | 224 | 0.0012 | 0.016 |
|  | GO:0003824 | F | catalytic activity | 76 | 14101 | 0.0015 | 0.018 |
|  | GO:0050662 | F | coenzyme binding | 8 | 604 | 0.0015 | 0.018 |
|  | GO:0009507 | C | chloroplast | 43 | 2811 | 1.70E-13 | 3.70E-11 |
|  | GO:0009536 | C | plastid | 43 | 2854 | 2.70E-13 | 3.70E-11 |
|  | GO:0009532 | C | plastid stroma | 20 | 618 | 5.10E-13 | 4.70E-11 |
|  | GO:0009570 | C | chloroplast stroma | 19 | 599 | 2.50E-12 | 1.80E-10 |
|  | GO:0044435 | C | plastid part | 25 | 1324 | 1.00E-10 | 5.50E-09 |
|  | GO:0044434 | C | chloroplast part | 23 | 1306 | 1.70E-09 | 8.00E-08 |
|  | GO:0048046 | C | apoplast | 15 | 618 | 1.40E-08 | 5.40E-07 |
|  | GO:0044444 | C | cytoplasmic part | 70 | 9427 | 6.50E-07 | 2.20E-05 |
|  | GO:0005737 | C | cytoplasm | 80 | 11818 | 3.60E-06 | 0.00011 |
|  | GO:0005777 | C | peroxisome | 8 | 267 | 5.90E-06 | 0.00015 |
|  | GO:0042579 | C | microbody | 8 | 267 | 5.90E-06 | 0.00015 |
|  | GO:0009579 | C | thylakoid | 10 | 572 | 4.70E-05 | 0.0011 |
|  | GO:0005576 | C | extracellular region | 20 | 2029 | 7.50E-05 | 0.0016 |
|  | GO:0055035 | C | plastid thylakoid membrane | 8 | 421 | 0.00014 | 0.0026 |
|  | GO:0009535 | C | chloroplast thylakoid membrane | 8 | 421 | 0.00014 | 0.0026 |
|  | GO:0042651 | C | thylakoid membrane | 8 | 438 | 0.00019 | 0.0032 |
|  | GO:0034357 | C | photosynthetic membrane | 8 | 449 | 0.00022 | 0.0036 |
|  | GO:0031976 | C | plastid thylakoid | 8 | 467 | 0.00029 | 0.0039 |
|  | GO:0044436 | C | thylakoid part | 8 | 466 | 0.00028 | 0.0039 |
|  | GO:0009534 | C | chloroplast thylakoid | 8 | 467 | 0.00029 | 0.0039 |
|  | GO:0031984 | C | organelle subcompartment | 8 | 543 | 0.00076 | 0.01 |
|  | GO:0046658 | C | anchored to plasma membrane | 5 | 237 | 0.0015 | 0.019 |
|  | GO:0044422 | C | organelle part | 39 | 6488 | 0.0022 | 0.025 |
|  | GO:0044446 | C | intracellular organelle part | 39 | 6476 | 0.0021 | 0.025 |
|  | GO:0009526 | C | plastid envelope | 8 | 661 | 0.0026 | 0.028 |
| group4 | GO:0006412 | P | translation | 5 | 1079 | 3.90E-04 | 1.80E-02 |

1. P: Biological Process C; Cellular Component F: Molecular Function
